# Femoral anteversion and tibial torsion only explain 25% of variance in regression analysis of foot progression angle in children with diplegic cerebral palsy

**DOI:** 10.1186/1743-0003-10-56

**Published:** 2013-06-15

**Authors:** Kyoung Min Lee, Chin Youb Chung, Ki Hyuk Sung, Tae Won Kim, Seung Yeol Lee, Moon Seok Park

**Affiliations:** 1 Department of Orthopaedic Surgery, Seoul National University Bundang Hospital, 300 Gumi-Dong, Bundang-Gu, Sungnam, Kyungki 463-707, South Korea; 2 Department of Orthopaedic Surgery, Kwandong University Myongji Hospital, 697-24 Hwajung-Dong, Dukyang-Gu, Goyang-Si, Kyungki 412-270, South Korea; 3 Department of Orthopaedic Surgery,Ulsan Hospital, 34-72 Shinjung-DongNam-Gu, Ulsan 680-742, South Korea

**Keywords:** Gait parameter, Torsional bony deformities, Cerebral palsy

## Abstract

**Background:**

The relationship between torsional bony deformities and rotational gait parameters has not been sufficiently investigated. This study was to investigate the degree of contribution of torsional bony deformities to rotational gait parameters in patients with diplegic cerebral palsy (CP).

**Methods:**

Thirty three legs from 33 consecutive ambulatory patients (average age 9.5 years, SD 6.9 years; 20 males and 13 females) with diplegic CP who underwent preoperative three dimensional gait analysis, foot radiographs, and computed tomography (CT) were included. Adjusted foot progression angle (FPA) was retrieved from gait analysis by correcting pelvic rotation from conventional FPA, which represented the rotational gait deviation of the lower extremity from the tip of the femoral head to the foot. Correlations between rotational gait parameters (FPA, adjusted FPA, average pelvic rotation, average hip rotation, and average knee rotation) and radiologic measurements (acetabular version, femoral anteversion, knee torsion, tibial torsion, and anteroposteriortalo-first metatarsal angle) were analyzed. Multiple regression analysis was performed to identify significant contributing radiographic measurements to adjusted FPA.

**Results:**

Adjusted FPA was significantly correlated with FPA (r=0.837, p<0.001), contralateral FPA (r=0.492, p=0.004), pelvic rotation during gait (r=−0.489, p=0.004), knee rotation during gait (r=0.376, p=0.031), and femoral anteversion (r=0.350, p=0.046). In multiple regression analysis, femoral anteversion (p=0.026) and tibial torsion (p=0.034) were found to be the significant contributing structural deformities to the adjusted FPA (R^2^=0.247).

**Conclusions:**

Femoral anteversion and tibial torsion were found to be the significant structural deformities that could affect adjusted FPA in patients with diplegic CP. Femoral anteversion and tibial torsion could explain only 24.7% of adjusted FPA.

## Background

Rotational gait problem is a common problem in patients with cerebral palsy, and compromises gait efficiency and function [1]. It is known to be caused by abnormal femoral anteversion and abnormal tibial torsion and clinical practice is usually focused on these two structures in the processes of evaluation and treatment. It is supported by a recent study, which reported the most common causes of intoeing gait in patients with diplegic cerebral palsy are internal rotation of hip and internal tibial torsion [2]. However, degree of femoral anteversion and tibial torsion do not necessarily reflect the severity of the rotational gait problem, which implies that intoeing might not be sufficiently explained by torsional bony deformities of the femur and tibia.

The physical examinations conducted to evaluate torsional bony deformities of the lower extremities include hip rotation angle for the assessment of femoral anteversion, thigh foot angle for tibial torsion, and foot shape. More recently, three dimensional (3D) gait analyses has been used to detect dynamic rotational deviations more accurately during gait. Furthermore, computed tomography (CT) is considered to be the gold standard for the evaluation of static torsional bony deformities of femur and tibia [3,4].

Although several authors have investigated the relationship between torsional bony deformities and rotational gait deviations, the majority have focused on deformities of femur and tibia [1,5-8]. However, various types of bony deformities, such as, pelvic rotation, acetabular torsion, knee joint torsion between femur and tibia, ankle joint torsion between the bimalleolar axis and talus, and foot deformity including forefoot adduction and abduction, could affect overall rotational alignment. In addition, spinal deformities, trunk balance, and balance between external and internal rotator muscles could affect rotational gait deviations.

We hypothesize that there might be torsional bony or structural deformities other than femoral anteversion and tibial torsion, affecting the rotational gait deviations. This study was undertaken to investigate the importance of torsional structural deformities in rotational gait parameters in patients with diplegic cerebral palsy.

## Methods

This retrospective study was approved by the institutional review board at our hospital (a tertiary referral center for cerebral palsy). Consecutive ambulatory patients with diplegic cerebral palsy underwent preoperative 3D gait analysis, physical examinations, and weight bearing anteroposterior foot radiographs, between October 2003 and December 2005. Of these, patients that showed abnormal rotational profile (foot progression angle on visual inspection, hip internal rotation, hip external rotation, thigh-foot angle, shape of foot lateral border) on physical examinations or abnormal rotational gait parameters on 3D gait analysis underwent CT examinations, and were included in this study. The exclusion criteria were previous history of fracture, infection, or any other conditions that could affect the anatomical structures. A total of 33 legs of 33 patients were included. Mean patient age was 9.5 years (SD 6.9 years), and there were 20 males and 13 females. Fifteen patients were of GMFCS level I and 18 were of GMFCS II.

3D gait analysis was performed using a Motion Analysis system (Motion Analysis, Santa Rosa, California) equipped with seven CCD cameras. Markers were placed as for the Helen Hayes marker set [9] by a single operator with 17 years of experience, who also performed data processing. Markers were attached to both anterior superior iliac spine, sacrum in the middle of the left and right posterior superior iliac spine, lateral thigh, medial and lateral femoral epicondyles, lateral shank, medial and lateral malleoli, heel, and dorsal foot between the 2^nd^ and 3^rd^ metatarsal heads. Medial femoral epicondyle and medial malleolar markers were detached during walking for dynamic data collection. Patients were asked to walk barefoot on a 9 m walkway three times and averaged kinematic data were recorded. From these, kinematic data of the lower extremity joint or segments were obtained in terms of sagittal, coronal, and transeverse planes at each 1% increment throughout the gait cycle. Rotational gait parameters on the transverse plane were retrieved and included in this study, which were foot progression angle, pelvic rotation, hip rotation, and knee rotation.

CT scans (Mx8000IDT; Philips Healthcare Korea, Seoul, South Korea) were acquired at a thickness of 1mm around the hip joint, knee joint, and ankle joint. CT was performed using a multidetector unit using age- and weight specific pediatric protocols to minimize radiation dose. The scanned region covered the acetabulum, femoral head and neck, femoral condyles, tibial condyles, and both malleoli. CT images were obtained using a picture archiving communication system (PACS) (IMPAX, Agfa Healthcare, Mortsel, Belgium) and measurements were performed using PACS software. CT scans were taken with the patients in a supine position without weight bearing. Weight bearing anteroposterior foot radiographs were taken using a UT 2000 unit (Philips, Eindhoven, the Netherlands) at a source to image distance of approximately 100 cm with 50 kVp and 5 mAs with a cassette hold device. Acetabular version, femoral anteversion, knee torsion, and tibial torsion were measured on CT images, and talo-first metatarsal angle was measured on foot radiographs.

### Gait kinematic parameters

Rotational kinematic data, namely, average foot progression angle instance, average hip rotation, average knee rotation, and average pelvic rotation, were obtained by 3-D gait analysis. Foot progression angle was the angle of the foot relative to the progressive direction of the subjects during gait. Hip rotation was the rotation of femur relative to the pelvic segment. Knee rotation was the rotation angle of tibia relative to the femoral segment, which is different from the usual definition of offset by static knee rotation. Pelvic rotation was the rotation angle of pelvic segment relative to the progressive direction of the subject during gait. Adjusted foot progression angle was used to correct for the effect of pelvic rotation because this can be affected by spinal deformity or trunk muscle imbalance. Adjusted foot progression angle was defined and calculated by subtracting pelvic rotation from the foot progression angle. This angle was calculated at each 1% increment through the gait cycle. The adjusted foot progression angle represents the angle between the tip of the femoral head and the foot, which is the rotation of the lower extremity. Foot progression angle of the contralateral side was also calculated.

### Consensus building and the interobserver reliabilities of radiologic measurements

Four orthopaedic surgeons with 12, 10, 8, and 5 years of orthopaedic experience held consensus building sessions for CT and radiographic measurements, and two of these with 10 and 8 years of experience measured radiographic indices for interobserver reliability determinations. Interobserver reliability was evaluated using intraclass correlation coefficients [10] and mean absolute differences [11].

**Figure 1:**
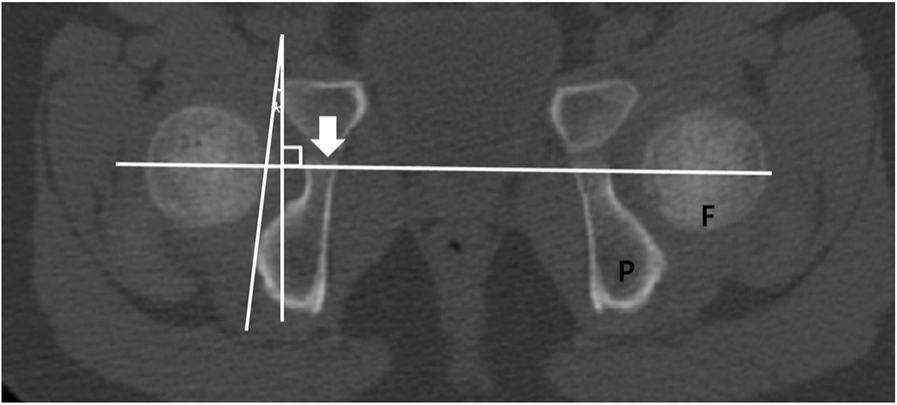
Acetbular version (asterisk) is the angle between a line connecting the anterior and posterior acetabular bony margin and another line perpendicular to a line connecting triradiate cartilage (arrow) within pelvic bone (P) or centers of bilateral femoral heads (F).

**Figure 2:**
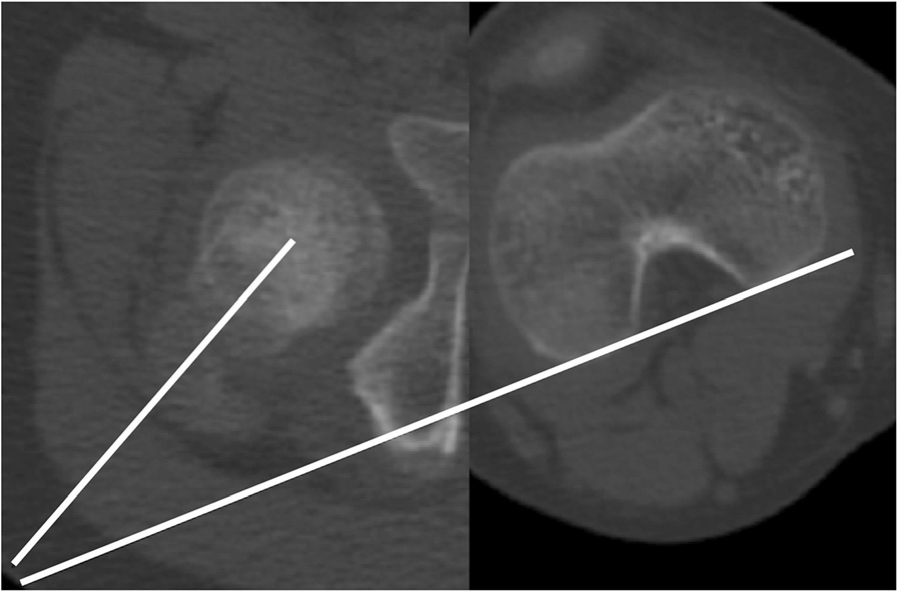
Femoral anteversion is the angle between a line connecting the centers of femoral head and neck, and another line connecting the posterior margin of medial and lateral femoral condyles.

**Figure 3:**
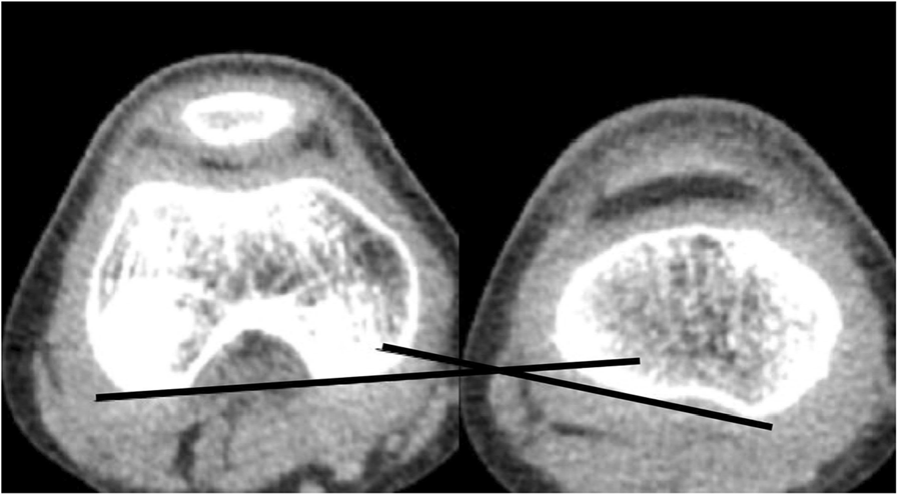
Knee torsion is the angle between a line connecting the posterior margin of medial and lateral femoral condyles and another line connecting the posterior margin of medial and lateral tibial condyles.

**Figure 4:**
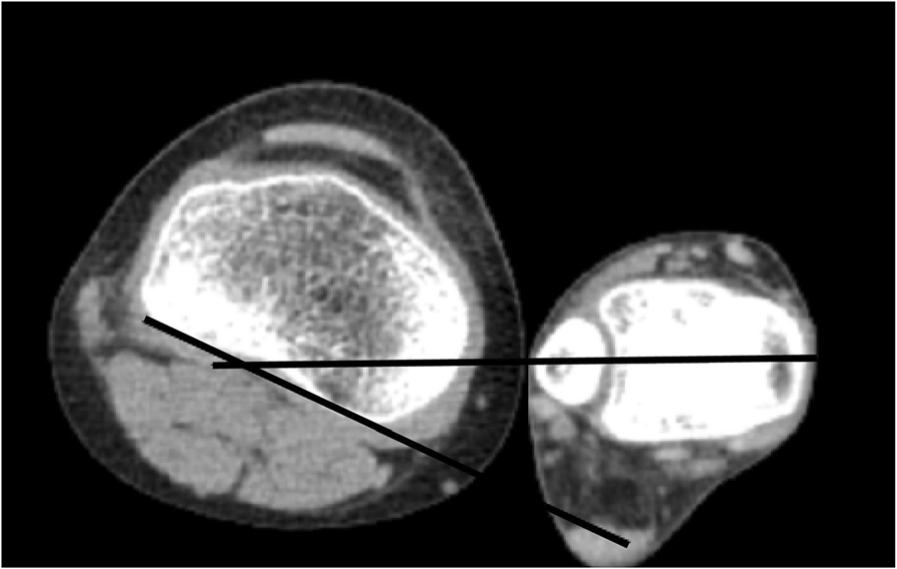
Tibial torsion is the angle between a line connecting the posterior margin of medial and lateral tibial condyles and another line connecting the tips of medial and lateral malleoli.

**Figure 5:**
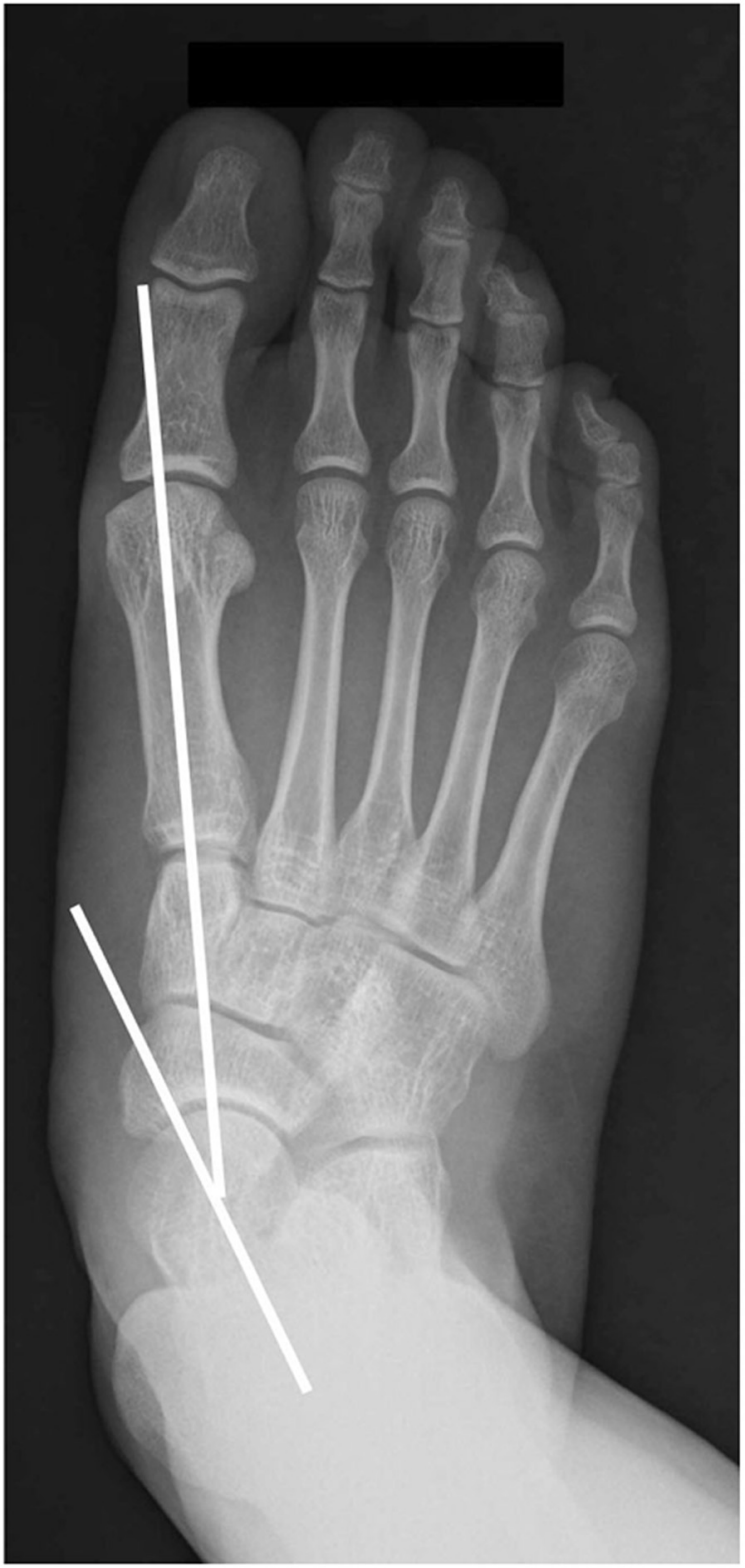
Talo-first metatarsal angle is the angle between the longitudinal axis of talus and that of first metatarsal bone on weight bearing anteroposterior foot radiograph.

On axial CT images, acetabular version was defined as the angle between a line connecting the anterior and posterior acetabular bony margin and another line perpendicular to a line connecting the triradiate cartilages or centers of bilateral femoral heads [12,13] (Figure 1). Femoral anteversion was defined as the angle between a line connecting the centers of the femoral head and neck, and another line connecting the posterior margins of the medial and lateral femoral condyles [14] (Figure 2). Knee torsion was defined as the angle between a line connecting the posterior margins of the medial and lateral femoral condyles and another line connecting the posterior margins of the medial and lateral tibial condyles (Figure 3). Tibial torsion was defined as the angle between a line connecting the posterior margins of the medial and lateral tibial condyles and another line connecting the tips of medial and lateral malleoli [4] (Figure 4). On weight bearing anteroposterior foot radiographs, the talo-first metatarsal angle was defined as the angle between the longitudinal axis of the talus and that of the first metatarsal bone [15] (Figure 5).

### Relations between CT and radiographic indices, and torsional gait parameters

Correlations between structural torsional deformities (measured on CT and radiographic images) and torsional gait parameters were calculated using Pearson’s correlation coefficients.

### Multiple regression analysis

Multiple regression analysis was performed to identify which structural torsional deformities significantly contributed to adjusted foot progression angle. Independent variables included; acetabular version, femoral anteversion, knee torsion, tibial torsion, and anteroposterior talo-first metatarsal angle.

### Statistical methods

This study used intraclass correlation coefficients (ICCs) for reliability testing [10]. The required sample size for reliability testing of CT and radiographic measurements was calculated beforehand to determine the minimum number of patients required. The target value of ICCs for CT and radiographic measurement was 0.9 and a 95% confidence interval of 0.2. The sample size was calculated using Bonnett’s approximation [16] to be 15 patients for two observers. Right legs were selected for statistical independence, and included for data analysis [17].

Descriptive statistics were used to summarize patients’ demographics, CT and radiographic measurements, and gait parameters. Data normality was determined using the Kolmogorov-Smirnov test. Interobserver reliability of CT and radiographic measurements was analyzed using mean absolute difference (MAD) as well as intraclass correlation coefficients (ICCs). ICCs and their 95% confidence intervals were determined in the setting of a two-way random effect model, a single measurement, and absolute agreement. Correlations between CT and radiographic measurements, and rotational gait parameters were analyzed using Pearson’s correlation coefficients. Multiple regression analysis was performed to identify structural torsional deformities (CT and radiographic measurements) that contributed significantly to adjusted foot progression angle. Goodness of fit is presented using adjusted R^2^ values. Statistical significance was accepted for p values of < 0.05.

## Results

Average foot progression angle in stance was 3.9° (SD 11.2), average femoral anteversion was 24.5° (SD 6.4), and average tibial torsion was 18.2° (SD 8.2) (Table 1). Anteroposterior talo-first metatarsal angle measured on weight bearing foot radiographs showed the highest reliability with an ICC value of 0.967, followed by tibial torsion measured on axial CT images (ICC 0.887), and knee torsion (ICC 0.684). In terms of mean absolute difference, tibial torsion and anteroposterior talo-first metatarsal angle showed lowest measurement errors, followed by acetabular version (mean absolute difference 2.6°) and knee torsion (mean absolute difference 5.4°) (Table 2).

**Table 1:** Patients’ demographics and data

	**Age**	**Gender**	**GMFCS**	**Kinematic parameters**					**Radiologic measurements**				
				**FPA in**	**Pelvic**	**Hip**	**Knee**	**Adjusted**	**Acetabular**	**Femoral**	**Knee**	**Tibial**	**Talo-1**^**st**^**MT**
				**stance (°)**	**rotation (°)**	**rotation (°)**	**rotation(°)**	**FPA (°)**	**version (°)**	**anteversion (°)**	**torsion (°)**	**torsion (°)**	**angle (°)**
1	6.0	F	II	−6.77	−6.61	15.42	0.39	−0.16	7.25	46.30	12.90	26.30	10.80
2	12.3	M	I	−4.05	3.44	5.79	8.30	−7.48	13.80	28.10	12.10	33.90	17.30
3	13.3	M	I	7.16	9.78	−3.18	−5.22	−2.62	8.60	25.40	7.70	13.90	23.30
4	7.1	F	I	−4.60	−5.82	2.50	−15.29	1.22	15.80	31.00	4.20	15.00	20.40
5	23.8	M	II	7.98	8.93	−1.27	1.65	−0.95	13.90	25.40	−5.90	19.10	14.60
6	6.3	F	II	16.32	−5.25	−5.39	20.00	21.57	15.60	33.90	12.20	24.00	15.60
7	5.5	M	I	25.63	−1.45	18.45	21.40	27.08	18.70	41.10	15.50	23.60	3.40
8	8.8	M	II	0.72	−4.57	21.40	6.04	5.28	11.50	46.00	4.50	20.10	21.80
9	16.7	F	II	−0.86	3.16	5.68	−4.19	−4.02	11.80	26.40	-	23.30	−23.70
10	10.0	M	I	−9.85	8.87	−6.81	−11.85	−18.72	13.20	22.10	1.10	27.80	9.30
11	5.8	M	II	−2.12	−13.45	6.66	−4.32	11.33	15.10	37.00	14.70	17.60	29.60
12	5.2	M	II	11.85	6.42	−5.92	−9.60	5.43	5.10	24.60	24.60	23.50	4.10
13	6.1	F	II	11.22	1.97	−1.11	−6.85	9.25	8.80	55.40	5.40	17.90	4.70
14	5.8	F	I	11.07	−6.14	19.64	−5.80	17.21	10.10	46.60	1.80	19.80	21.80
15	5.3	M	II	22.21	7.63	7.49	−2.78	14.59	10.50	53.30	13.40	23.80	1.90
16	5.8	M	I	22.83	8.69	4.14	8.10	14.14	13.50	56.60	0.30	31.40	−4.60
17	5.8	F	I	−14.36	1.94	7.07	−7.88	−16.30	9.70	35.50	7.10	31.50	-
18	5.9	M	I	−4.98	6.95	5.09	7.15	−11.93	11.40	46.80	−3.00	6.30	−22.40
19	8.0	M	I	5.18	−3.20	11.97	8.19	8.38	14.90	41.20	3.50	21.10	−26.10
20	6.7	F	I	0.63	1.29	0.09	−1.62	−0.66	11.90	31.20	0.50	25.60	15.60
21	10.0	M	II	−2.31	8.95	3.99	−11.58	−11.26	11.80	32.50	8.20	34.40	35.60
22	7.0	F	II	11.85	1.71	15.23	−3.57	10.14	19.20	52.90	8.40	23.30	−1.50
23	6.8	M	I	13.22	−19.17	19.18	7.84	32.39	10.40	53.80	2.00	22.50	−1.70
24	5.5	M	I	−18.27	−8.13	−16.09	3.00	−10.14	12.60	51.60	11.60	34.40	-
25	37.6	F	I	−18.29	−1.96	3.34	7.93	−16.32	10.90	32.20	10.80	33.50	5.70
26	5.7	M	I	−0.62	−2.48	−12.49	−1.66	1.86	19.70	32.50	12.30	21.60	11.50
27	9.6	M	II	4.12	7.61	−10.26	−0.17	−3.48	7.90	32.90	8.40	3.70	7.50
28	9.6	M	II	20.58	−6.20	−1.11	8.39	26.78	10.50	37.90	3.40	−0.12	15.10
29	19.3	F	II	7.22	1.03	−4.90	2.79	6.19	10.60	58.90	5.80	6.90	4.62
30	11.6	F	II	5.71	2.38	−0.41	0.74	3.32	10.00	15.60	9.40	19.30	16.70
31	9.5	M	II	7.97	3.88	10.55	−20.43	4.08	11.40	34.40	−2.30	26.40	−5.40
32	10.7	M	II	5.17	−8.29	3.97	−10.10	13.46	16.80	39.00	3.90	14.30	1.30
33	15.9	F	II	−4.09	−0.34	18.99	−27.85	−3.75	18.40	60.00	-	42.20	9.90
Data summary	9.5 (6.9)	M:F = 20:13	I/II = 15/18	3.9 (11.2)	0.05 (7.0)	4.2 (9.7)	−1.2 (10.4)	3.8 (12.8)	12.5 (3.5)	39.0 (11.8)	6.9 (6.4)	22.1 (9.4)	7.6 (14.3)

*GMFCS* gross motor function classification system, *FPA* foot progression angle; Talo-1^st^ MT angle, anteroposterior talo-first metatarsal angle.

Positive values indicate outtoeing or external rotation for kinematic parameters.

Positive values indicate external rotation for radiologic measurements.

-, data unavailable due to poor quality of image.

**Table 2:** Interobserver reliabilities of radiographic measurements

	**Acetabular version**	**Femoral anteversion**	**Knee torsion**	**Tibial torsion**	**Talo-1**^**st**^**MT**
ICC	0.426	0.427	0.684	0.887	0.967
(95% CI)	(0.031 to 0.752)	(0.045 to 0.756)	(0.077to 0.914)	(0.644 to 0.963)	(0.919 to 0.987)
MAD	2.6° (SD 3.0)	9.7° (SD 5.4)	5.4° (SD 3.8)	2.5° (SD 2.2)	2.5° (SD 2.2)

Talo-1^st^ MT, anteroposterior talo-first metatarsal angle.

*MAD* mean absolute difference, *SD* standard deviation.

Foot progression angle was found to be significantly correlated with adjusted foot progression angle (r=0.837, p<0.001) and contralateral foot progression angle (r=0.424, p=0.014). Increased femoral anteversion was found to be significantly correlated with internal hip rotation during gait (r=0.385, p=0.027), whereas the correlation between internal tibial torsion and external knee rotation during gait was not significant (r=−0.214, p=0.232). Adjusted foot progression angle was found to be significantly correlated with contralateral foot progression angle (r=0.492, p=0.004), external pelvic rotation during gait (r=−0.489, p=0.004), external knee rotation during gait (r=0.376, p=0.031), and decreased femoral anteversion (r=0.350, p=0.046) (Table 3).

**Table 3:** Correlation between CT and radiographic measurements, and rotational gait parameters

	**FPA**	**Contralat**	**Pelvic**	**Hip**	**Knee**	**Adjusted**	**Acetabular**	**Femoral**	**Knee**	**Tibial**
		**FPA**	**rotation**	**rotation**	**rotation**	**FPA**	**version**	**anteversion**	**torsion**	**torsion**
Contralat	**0.424**									
FPA	**(0.014)**									
Pelvic	0.068	−0.221								
rotation	(0.707)	(0.218)								
Hip	0.190	−0.064	−0.300							
rotation	(0.289)	(0.722)	(0.090)							
Knee	0.307	0.256	−0.197	−0.024						
rotation	(0.083)	(0.150)	(0.273)	(0.894)						
Adjusted	**0.837**	**0.492**	**−0.489**	0.331	**0.376**					
FPA	**(<0.001)**	**(0.004)**	**(0.004)**	(0.060)	**(0.031)**					
Acetabular	0.045	−0.049	−0.211	0.152	0.039	0.155				
version	(0.802)	(0.785)	(0.239)	(0.399)	(0.831)	(0.388)				
Femoral	0.219	0.199	−0.290	**0.385**	0.052	**0.350**	0.132			
anteversion	(0.221)	(0.268)	(0.102)	**(0.027)**	(0.772)	**(0.046)**	(0.464)			
Knee	−0.003	0.178	−0.111	−0.155	0.135	0.059	−0.071	−0.117		
torsion	(0.988)	(0.338)	(0.553)	(0.407)	(0.469)	(0.753)	(0.706)	(0.532)		
Tibial	−0.344	−0.018	0.056	0.222	−0.214	−0.332	0.219	0.059	0.228	
torsion	(0.050)	(0.921)	(0.758)	(0.215)	(0.232)	(0.059)	(0.220)	(0.743)	(0.218)	
AP talo-1^st^	−0.120	0.178	−0.099	−0.127	−0.150	−0.042	−0.039	−0.255	0.238	0.083
MT	(0.520)	(0.339)	(0.595)	(0.497)	(0.419)	(0.822)	(0.836)	(0.167)	(0.215)	(0.656)

*FPA* foot progression angle; AP talo-1^st^ MT, anteroposterior talo-first metatarsal angle.

Statistical signifance (p<0.05) was presented as bold characters.

By multiple regression analysis, femoral anteversion (p=0.026) and tibial torsion (p=0.034) were found to be significant structural deformities contributing to the adjusted foot progression angle (R^2^=0.247) (Table 4). When the conventional foot progression angle was the dependent variable in the multiple regression analysis, the R^2^ value was 0.176.

**Table 4:** Multiple regression analysis to identify significantly contributing structural factors to adjusted foot progression angle

	**Standardized**	**95% CI**	**p-value**	**Standardized**	**95% CI**	**p-value**
	**Beta**			**Beta**		
Acetabular version	0.217	−0.5 to 2.0	0.222	*	*	*
Femoral anteversion	**0.496**	**0.1 to 1.0**	**0.014**	**0.371**	0.1 to 0.8	**0.026**
Knee torsion	−0.247	−0.3 to 1.1	0.178	*	*	*
Tibial torsion	0.218	−0.9 to 0.2	0.236	**−0.354**	**−0.9 to −0.1**	**0.034**
AP talo-1MT	0.083	−0.3 to 0.4	0.665	*	*	*

R^2^ = 0.336 R^2^ = 0.247 FPA, foot progression angle; AP talo-MT, anteroposterior talo-first metatarsal angle.

* Variables not included in multiple regression analysis.

Statistical signifance (p<0.05) was presented as bold characters.

## Discussion

Rotational gait deviations are important when treating patients with diplegic cerebral palsy, and although they are usually treated by rotation osteotomy, the effects of structural torsional bony deformity on rotational gait parameters have not been sufficiently investigated. In this study, relationships between torsional bony deformities, represented by CT and radiographic measurements, and rotational gait parameters were examined, and the degree of contribution of torsional structural deformities to the rotational gait parameters were evaluated. Adjusted foot progression angle, which corrects for pelvic rotation excluding the effect of spinal deformity or trunk balance, appeared to be more relevant to clinical use than the conventional foot progression angle when evaluating the torsional deformities of lower extremities. The adjusted foot progression angle was theoretically to represent the rotational gait parameters between the tip of the femoral head and the foot, which is essentially the lower extremity. We believe this angle is more clinically relevant to clinical practice, considering the evaluation and treatment of the rotational gait deviation because current clinical practice is focused on the lower extremity, including the femur and the tibia. In multiple regression analysis, femoral anteversion and tibial torsion were found to be the structural deformities that significantly affected the adjusted foot progression angle in patients with diplegic cerebral palsy with R^2^ of 0.247.

The conventional foot progression angle did not show significant correlations with clinically measurable structural deformities and rotational gait parameters. Therefore, factors other than torsional deformity of the lower extremity, such as, spinal deformity and trunk balance, might need to be considered when evaluating the conventional foot progression angle, and most importantly, selective motor control and other neuro-muscular factors need to be further investigated. Multiple regression analysis results also suggested that the adjusted foot progression angle was more relevant than the conventional foot progression angle because the former were more likely explained by the currently measurable structural deformities in a clinical viewpoint.

In the present study, foot progression angle and adjusted foot progression angle showed significant correlations with contralateral foot progression angle. Foot progression angle is necessarily affected by contralateral rotational gait parameters because the lower extremities of both sides are interlinked through the pelvis. Therefore, in the clinical situation, although we correct structural rotational deformity of the lower extremity, if rotational deformity of the contralateral lower extremity or the upper level of the rotational deformity, such as, of the pelvis and spine, has not been corrected, the torsional gait problem will not be resolved. These complex relationships could be the reason why clinicians sometimes might not achieve the intended surgical outcome. In addition, imbalance between the external rotator and internal rotator muscles as well as the movement or deformities in other planes could affect rotational gait deviations [8], and this need to be considered when treating rotational gait problems.

According to multiple regression analysis, femoral anteversion (p=0.026) and tibial torsion (p=0.034) significantly affected adjusted foot progression angle. This result supports current consensus and practice, because femoral derotation osteotomy and tibial derotation osteotomy comprise the mainstream surgical treatment of torsional problems in patients with diplegic cerebral palsy. However, femoral anteversion and tibial torsion explained only 24.7% of adjusted foot progression angle. Furthermore, although all structural components were included in the multiple regression analysis, they explained only 33.6% of adjusted foot progression angle (Table 4). Therefore, factors other than structural torsional deformities of the lower extremity, such as, spinal deformity, trunk balance, and rotator muscle balance, probably contribute to rotational gait problems, and thus, these factors should be considered clinically.

This study has some limitations that should be addressed before discussing the results. First, not all components of structural torsional deformity were included. In particular, torsional deformity of the ankle joint, which could be represented by the angle between the bimalleolar axis and the longitudinal axis of talus, could not be evaluated because no weight bearing CT scan was available. Spinal deformity and trunk muscle imbalance, both of which could affect pelvic rotation, were neither directly measured nor included. Further study is needed to investigate the effect of pelvic and spinal motion on the rotational gait parameters more in detail, including the kinematic data of trunk motion and the effect of upper body brace. Second, the study population was confined to diplegic cerebral palsy patient that were able to walk, and thus, the study results cannot be generalized to all cerebral palsy patients. Third, degrees of ossification in bony structures depend on patient age, which might have affected CT and radiographic measurements, and caused results bias. Fourth, this study included from young children to older teenagers. The gait of young children is known to be different from that of teenagers, and the wide range of age might have biased the study results. Fifth, movements and deformities in the sagittal and coronal planes can influence rotations in the transverse plane [8] but this study did not consider the movement in the other planes besides that of the transverse plane. Sixth, although knee torsion measured the soft tissue property between distal femur and proximal tibia, most of the structural deformities focused on bony structures in this study. However, we believe that bony deformities are more reliabile and valid than soft tissue balance, in measuring torsional malalignment.

A previous study showed that structural torsional deformity was significantly correlated with rotational gait parameters in tibia but not in femur, which is different from our results [5]. We believe that the different result might have been caused by the different patients’ age, degree of ossification affecting radiologic measurements, and different characteristics (functional level, uni- or bilateral involvement). Another study suggested that increased hip internal rotation during gait was associated with other factors besides increased femoral anteversion [8]. Although the study focused on only hip joint and the design was different from ours, the study showed that unpredictable anatomical factors could affect the rotational gait parameters. This provides some explanation of our results that the femoral and tibial torsion could determine only 24.7% of the adjusted foot progression angle.

Both ICC and MAD were used for reliability test of CT and radiographic measurements in this study. Although ICC is a frequently used method for reliability testing, it can be affected by measurement range, as well as measurement error [10], which sometimes make the clinical interpretation difficult. On the other hand, MAD represents the absolute value of measurement error. These are why acetabular version showed somewhat low ICC value despite low measurement error (MAD). Therefore, both ICC and MAD have their own implication in evaluating reliability and could be compensatory to each other. Considering both ICC and MAD, tibial torsion and talo-first metatarsal angle were reliable measurement while the measurement of the femoral anteversion was less reliable.

## Conclusions

The adjusted foot progression angle, which corrects the effect of pelvic rotation, appeared to be clinically more relevant than foot progression angle in patients with diplegic cerebral palsy. Furthermore, femoral anteversion and tibial torsion were found to be the significant structural deformities that could affect adjusted foot progression angle. However, femoral anteversion and tibial torsion were found to explain only 24.7% of adjusted foot progression angle. Thus, other structural factors, such as, pelvic rotation, that could be affected by trunk muscle balance, spinal deformity, knee torsion, foot deformity, or balance between the external and internal rotators, as well as important functional problems, including selective motor control and other neuro-muscular factors need to be considered when evaluating and treating torsional problems in patients with diplegic cerebral palsy.

## Competing interests

The authors declare that they have no competing interests.

## Authors’ contributions

KML, CYC, MSP: Study concept and design. TWK, KHS, SYL: Acquisition of data. TWK, CYC, SYL, KHS: Analysis and interpretation of data. KML, MSP: Drafting of the manuscript. TWK, KHS, SYL: Statistical analysis. KML, CYC, MSP: Study supervision. All authors read and approved the final manuscript.

## References

[B1] Gage JR Schwartz MH Koop SE Novacheck TF The identification and treatment of gait problems in cerebral palsy 2009 London: Mac Keith Press

[B2] Rethlefsen SA Healy BS Wren TA Skaggs DL Kay RM Causes of intoeing gait in children with cerebral palsy J Bone Joint Surg Am 2006 88 2175 2180 10.2106/JBJS.E.01280 17015594

[B3] Hernandez RJ Tachdjian MO Poznanski AK Dias LS CT determination of femoral torsion Am J Roentgenol 1981 137 97 101 10.2214/ajr.137.1.97 6787898

[B4] Jakob RP Haertel M Stussi E Tibial torsion calculated by computerised tomography and compared to other methods of measurement J Bone Joint Surg Br 1980 62B 238 242 7364840 10.1302/0301-620X.62B2.7364840

[B5] Aktas S Aiona MD Orendurff M Evaluation of rotational gait abnormality in the patients cerebral palsy J Pediatr Orthop 2000 20 217 220 10739285

[B6] Carriero A Zavatsky A Stebbins J Theologis T Shefelbine SJ Correlation between lower limb bone morphology and gait characteristics in children with spastic diplegic cerebral palsy J Pediatr Orthop 2009 29 73 79 10.1097/BPO.0b013e31819224d 19098651

[B7] Stefko RM de-Swart RJ Dodgin DA Kinematic and kinetic analysis of distal derotational osteotomy of the leg in children with cerebral palsy J Pediatr Orthop 1998 18 81 87 9449107

[B8] O'Sullivan R Walsh M Hewart P Jenkinson A Ross LA O'Brien T Factors associated with internal hip rotation gait in patients with cerebral palsy J Pediatr Orthop 2006 26 537 541 10.1097/01.bpo.0000217727.93546.2b 16791076

[B9] Kadaba MP Ramakrishnan HK Wootten ME Measurement of lower extremity kinematics during level walking J Orthop Res 1990 8 3 383 392 10.1002/jor.1100080310 2324857

[B10] Shrout PE Fleiss JL Intraclass correlations: uses in assessing rater reliability Psychol Bull 1979 86 420 428 18839484 10.1037//0033-2909.86.2.420

[B11] Lomnicki ZA The standard error of Gini's mean difference Ann Math Statistics 1952 23 635 637 10.1214/aoms/1177729346

[B12] Yazici M Kandemir U Atilla B Eryilmaz M Rotational profile of lower extremities in bladder exstrophy patients with unapproximated pelvis: a clinical and radiologic study in children older than 7 years J Pediatr Orthop 1999 19 531 535 10413008 10.1097/00004694-199907000-00022

[B13] Perreira AC Hunter JC Laird T Jamali AA Multilevel measurement of acetabular version using 3-D CT-generated models: implications for hip preservation surgery Clin Orthop Relat Res 2011 469 552 561 10.1007/s11999-010-1567-2 20872104 PMC3018214

[B14] Murphy SB Simon SR Kijewski PK Wilkinson RH Griscom NT Femoral anteversion J Bone Joint Surg Am 1987 69 1169 1176 3667647

[B15] Vanderwilde R Staheli LT Chew DE Malagon V Measurements on radiographs of the foot in normal infants and children J Bone Joint Surg Am 1988 70 407 415 3346265

[B16] Bonett DG Sample size requirements for estimating intraclass correlations with desired precision Stat Med 2002 21 1331 1335 10.1002/sim.1108 12111881

[B17] Park MS Kim SJ Chung CY Choi IH Lee SH Lee KM Statistical consideration for bilateral cases in orthopaedic research J Bone Joint Surg Am 2010 92 1732 1737 10.2106/JBJS.I.00724 20660236

